# Gibberellin-Regulation and Genetic Variations in Leaf Elongation for Tall Fescue in Association with Differential Gene Expression Controlling Cell Expansion

**DOI:** 10.1038/srep30258

**Published:** 2016-07-26

**Authors:** Qian Xu, Sanalkumar Krishnan, Emily Merewitz, Jichen Xu, Bingru Huang

**Affiliations:** 1 National Engineering Laboratory for Tree Breeding, College of Biological Sciences and Technology, Beijing Forestry University, Beijing, 100083, China; 2 Department of Plant Biology and Pathology, Rutgers University, New Brunswick, NJ, 08901, United States of America; 3 Department of Crop Science, Michigan State University, East Lansing, MI, 48824, United States of America

## Abstract

Leaf elongation rate (LER) is an important factor controlling plant growth and productivity. The objective of this study was to determine whether genetic variation in LER for a fast-growing (‘K-31’), and a dwarf cultivar (‘Bonsai’) of tall fescue (*Festuca arundinacea*) and gibberellic acid (GA) regulation of LER were associated with differential expression of cell-expansion genes. Plants were treated with GA_3_, trinexapac-ethyl (TE) (GA inhibitor), or water (untreated control) in a hydroponic system. LER of ‘K-31’ was 63% greater than that of ‘Bonsai’, which corresponded with 32% higher endogenous GA_4_ content in leaf and greater cell elongation and production rates under the untreated control condition. Exogenous application of GA_3_ significantly enhanced LER while TE treatment inhibited leaf elongation due to GA_3_-stimulation or TE-inhibition of cell elongation and production rate in leaves for both cultivars. Real-time quantitative polymerase chain reaction analysis revealed that three α-expansins, one β-expansin, and three xyloglucan endotransglycosylase (XET) genes were associated with GA-stimulation of leaf elongation, of which, the differential expression of *EXPA4* and *EXPA7* was related to the genotypic variation in LER of two cultivars. Those differentially-expressed expansin and XET genes could play major roles in genetic variation and GA-regulated leaf elongation in tall fescue.

Leaf growth is the major determining factor contributing to shoot biomass and yield production^1^,^2^. Leaf elongation rate (LER) is genetically controlled and developmentally regulated and varies with leaf age, leaf position, and between fast-growing cultivars and slow-growing cultivars^3^,^4^, but it is also very sensitive to external factors^5^,^6^,^7^. Depending on the objectives of plant production, either fast-growing or slow-growing leaves may be desirable, selectable traits in plant improvement. For example, for perennial grass species, fast-growing species are desirable for the productivity of grasses in forage or natural grasslands while slow-growing traits are important for turf grasses requiring mowing^8^,^9^. Therefore, understanding the mechanisms controlling leaf elongation is critically important for genetic modification of plants for fast- or slow-growing habits through transformation or molecular breeding.

Leaf elongation is controlled by cell elongation and cell division rates^10^,^11^. Both of those processes are located in the base of the elongating leaf which is called the leaf elongation zone and enclosed by the sheaths of older leaves in grasses^12^. The relative importance of each cell process accounting for the variations in leaf elongation rate is also variable, depending on plant species and environmental factors. The LER may be determined by both of cell elongation and production rates in some grass species, such as tall fescue (*Festuca arundinacea*), while the variations in LER can also be due to mostly differences in cell production rate rather than cell elongation in some other grass species, such as *Poa compressa*, *P. annua*, and *P. trivialis*^13^. Volenec *et al*.^14^ found a high-LER cultivar (26 mm d^−1^) of tall fescue had 25% longer epidermal cells and 24% higher cell production rate than a low-LER cultivar (18 mm d^−1^), suggesting genotypic variation in LER related to both cell elongation and production rate^14^. The cellular and molecular factors accounting for the genetic variation in leaf elongation are still not well understood.

Cell elongation is controlled by cell extensibility, which is regulated by cell-wall loosening proteins and enzymes including expansin and xyloglucan endotransglycosylase (XET)^15^,^16^. Expansin is the primary factor in the cell wall that mediates pH-dependent wall loosening, which can disrupt the non-covalent binding between the cell wall polysaccharides, thereby allowing turgor-driven wall extension^17^. Expansins are encoded by a large gene family including two major types of α-expansins (EXPA) and β-expansins (EXPB)^18^,^19^, which play critical roles in regulating cell expansion in the leaf elongation zone^20^. Previous studies have identified many expansin genes in maize *(Zea mays*)^21^, rice (*Oryza sativa*)^22^, wheat (*Triticum aestivum*)^23^, meadow fescue (*F. pratensis*)^24^ and other species^25^,^26^,^27^, the expression levels of which were positively related to leaf and stem elongation. Goh *et al*.^28^ reduced the expression of several expansin genes in arabidopsis (*Arabidopsis thaliana*) using an inducible microRNA construct and found that the decreased expansin gene expression led to a repression of leaf growth^28^. Over-expression of an expansin gene cloned from rice (*OsEXPA4*) increased the coleoptile and mesocotyl length by up to 31 and 97%, respectively, while in the anti-sense seedlings, the length of them decreased by up to 28 and 43%, respectively^29^.

XET is another important protein located in cell wall that has been associated with cell elongation in various plant species^30^. Many previous studies demonstrated that XET activity and gene expression were positively correlated to the elongation rate of leaf blades in grasses, such as barley (*Hordeum vulgare*)^31^, meadow fescue^24^, and maize^32^. There are various members of expansins and XET genes, but the specific genes related to genetic variation in leaf elongation are not well documented.

In addition, hormones, such as gibberellins (GAs), are known to affect leaf elongation in many species, such as arabidopsis^33^, maize^34^ and rice^34^,^35^. Most semi-dwarf cultivars with lower leaf growth rate were found to have either less sensitivity to GA or reduced levels of endogenous GA^36^,^37^. Bultynck and Lambers^38^ examined the effects of GA_3_ and paclobutrazol, an inhibitor of GA biosynthesis, on two *Aegilops* species with contrasting leaf elongation rates and found that addition of GA_3_ increased leaf elongation rate of both *Aegilops* species via stimulating both cell elongation and division while paclobutrazol inhibited leaf elongation rate via repressing cell elongation and division^38^. Similar results were also reported in wheat^39^ and barley^40^. However, whether genetic variation and the effects of GA on the elongation of leaves are associated with changes in expansin and XET expression is not clear. Understanding cellular and molecular mechanisms underlying genetic variations and hormonal regulation of leaf elongation will provide further insights into strategies to develop plants with desirable traits of fast-growing or slow-growing phenotypes.

Tall fescue has wide genetic variation in leaf elongation rate, with cultivars of fast-growing or slow-growing (or dwarf-type) phenotypes widely used as forage and turf grasses, respectively^41^,^42^. The various growth habits make tall fescue a good model species for studying mechanisms controlling leaf elongation in perennial grasses. In this study, it is hypothesized that the genetic variation in leaf elongation between fast-growing and dwarf-type tall fescue cultivars could be regulated by differential responses to GA, endogenous production of GA, and/or differential expression of cell-wall loosening genes controlling cell elongation. Therefore, the objectives of this study were to determine GA-regulation of leaf elongation and differential expression of several expansin and XET genes associated with the genetic variations in leaf elongation rate by comparing a fast-growing cultivar ‘K-31’ and a dwarf-type cultivar ‘Bonsai’.

## Results

### Differential leaf elongation rate between cultivars

**Figure 1:**
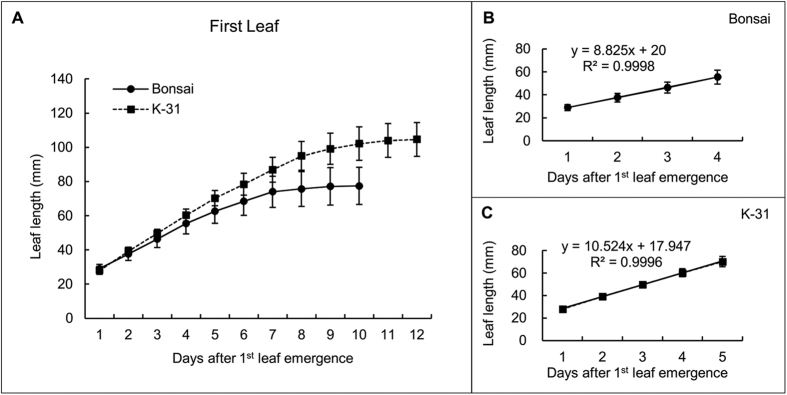
Elongation rates of the first leaf (youngest leaf of a plant) in cultivar ‘K-31’ and ‘Bonsai’. (**A**) The first leaf length of both cultivars in the elongating phase during 12-d emergence. The vertical bar is the standard error of mean leaf length (n = 40 replicates) at each given day of leaf emergence. (**B**) Changes of the first leaf length during the linear growth phase within the first 4 d of leaf emergence for ‘Bonsai’. (**C**) Changes of the first leaf length during the linear growth phase within the first 5 d of leaf emergence for ‘K-31’. The slope of the linear regression line represents leaf elongation rate (mm d^−1^) in (**B**) and (**C**). The function y = mx + b represents the linear relationship of leaf length (y) to days of leaf elongation (x) and the LER (m) was calculated by the equation m = [n∑(xy) − ∑x∑y]/[n∑(x^2^) − (∑x)^2^]. The R^2^ is the square of the correlation coefficient.

**Figure 2:**
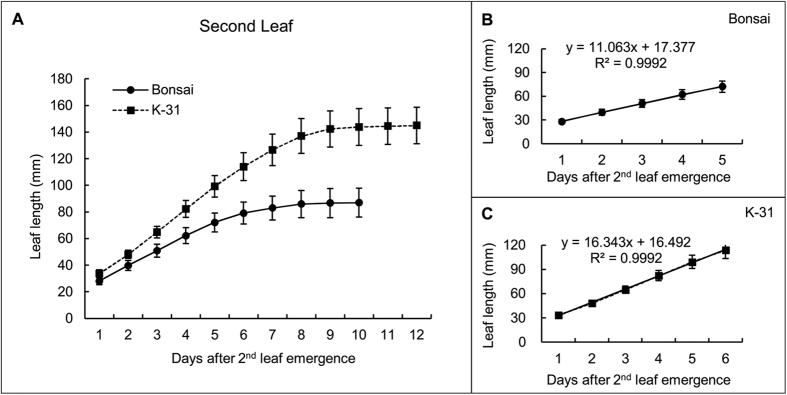
Elongation rate of the second leaf (second youngest leaf of a plant) in ‘K-31’ and ‘Bonsai’. (**A**) The second leaf length of both cultivars in the elongating phase during 12-d emergence. The vertical bar is the standard error of mean leaf length (n = 40 replicates) at each given day of leaf emergence. (**B**) Changes of the second leaf length during the linear growth phase within the first 5 d of leaf emergence for ‘Bonsai’. (**C**) Changes of the second leaf length during the linear growth phase within the first 6 d of leaf emergence for ‘K-31’. The slope of the linear regression line represents leaf elongation rate (mm d^−1^) in (**B**) and (**C**). The function y = mx + b represents the linear relationship of leaf length (y) to days of leaf elongation (x) and the LER (m) was calculated by the equation m = [n∑(xy) − ∑x∑y]/[n∑(x^2^) − (∑x)^2^]. The R^2^ is the square of the correlation coefficient.

**Figure 3:**
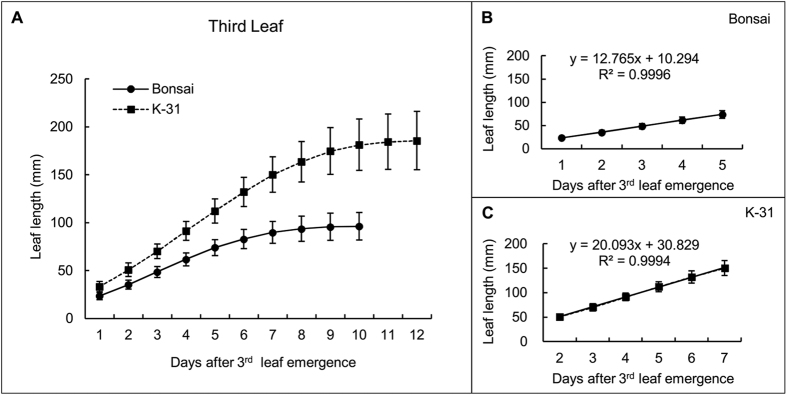
Elongation rate of the third leaf (third youngest leaf of a plant) in ‘K-31’ and ‘Bonsai’. (**A**) The third leaf length of both cultivars in the elongating phase during 12-d emergence. The vertical bar is the standard error of mean leaf length (n = 40 replicates) at each given day of leaf emergence. (**B**) Changes of the third leaf length during the linear growth phase within the first 5 d of leaf emergence for ‘Bonsai’. (**C**) Changes of the third leaf length during the linear growth phase within the first 6 d of leaf emergence for ‘K-31’. The slope of the linear regression line represents leaf elongation rate (mm d^−1^) in (**B**) and (**C**). The function y = mx + b represents the linear relationship of leaf length (y) to days of leaf elongation (x) and the LER (m) was calculated by the equation m = [n∑(xy) − ∑x∑y]/[n∑(x^2^) − (∑x)^2^]. The R^2^ is the square of the correlation coefficient.

Leaves of ‘K-31’ and ‘Bonsai’ exhibited differential elongation rate, and the differences became more pronounced with leaf age. The first leaf elongation rate of ‘K-31’ (10.52 mm d^−1^) was 19% higher than ‘Bonsai’ (8.82 mm d^−1^) (Fig. 1A–C); the second leaf elongation rate of ‘K-31’ (16.34 mm d^−1^) was 48% greater than ‘Bonsai’ (11.06 mm d^−1^) (Fig. 2A–C); and the third leaf was 57% greater in ‘K-31’ (20.09 mm d^−1^) than ‘Bonsai’ (12.77 mm d^−1^) (Fig. 3A–C).

**Figure 4:**
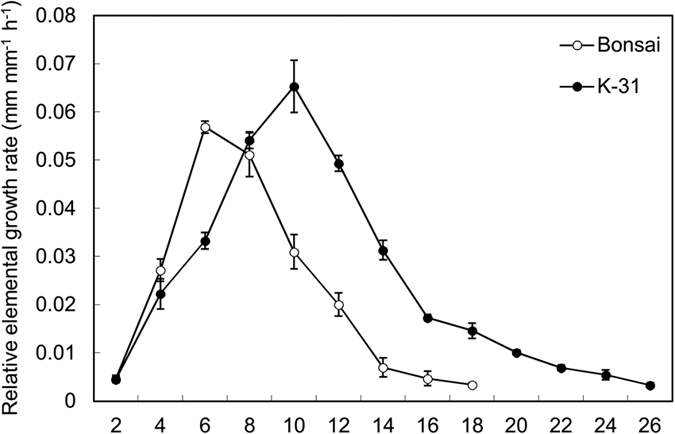
Spatial distribution of the relative elemental growth rate of the third leaf in a plant of ‘K-31’ and ‘Bonsai’. The vertical bar is the standard error of mean (n = 40 replicates) at each given distance from the leaf base.

The REGR along the third leaf was compared between the two cultivars (Fig. 4). The maximum REGR of ‘K-31’ was 14% higher than ‘Bonsai’. The length of elongation zone was also longer in ‘K-31’ compared with ‘Bonsai’, as ‘Bonsai’ leaf reached to the maximum elongation rate within 6 mm from the leaf base while ‘K-31’ leaves did not increase to the peak rate until 10 mm from the leaf base and maintained significantly greater rate than ‘Bonsai’ beyond 10 mm from the leaf base.

### Cultivar variations and exogenous GA application in endogenous GA content

**Figure 5:**
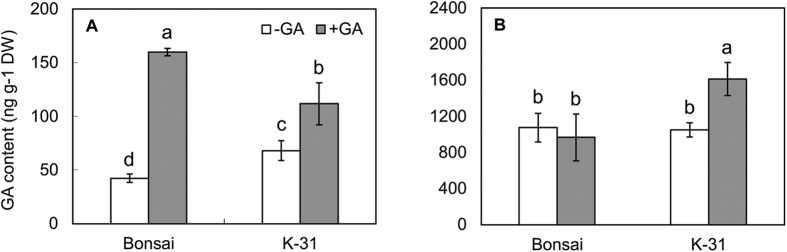
Endogenous GA_4_ (**A**) and GA_1_ (**B**) content of the third leaves of ‘K-31’ and ‘Bonsai’ with and without GA (50 μmol L^−1^ GA_3_) application. The vertical bar is the standard error of mean (n = 4 replicates of GA treatment, and each replicate contains at least 10 seedlings). Columns marked with different letters indicate significant differences between treatments and between cultivars based on LSD test (*P* = 0.05).

To investigate whether differences in LER could be related to GA levels, endogenous GA_1_ and GA_4_ contents of leaves were compared between the two cultivars with or without exogenous GA treatment. ‘K-31’ leaves had significantly higher endogenous GA_4_ level than ‘Bonsai’ leaves but there were no significantly differences in GA_1_ contents between those two genotypes (Fig. 5). The endogenous GA_4_ contents of leaves increased 3.77 fold and 1.64 fold by exogenous application of GA in ‘K-31’ and ‘Bonsai’, respectively. The endogenous GA_1_ content of leaves kept the same level in ‘Bonsai’ after GA application and increased by 54% in ‘K-31’.

### Effects of exogenous GA and TE application on leaf elongation, cell elongation, and cell division

**Figure 6:**
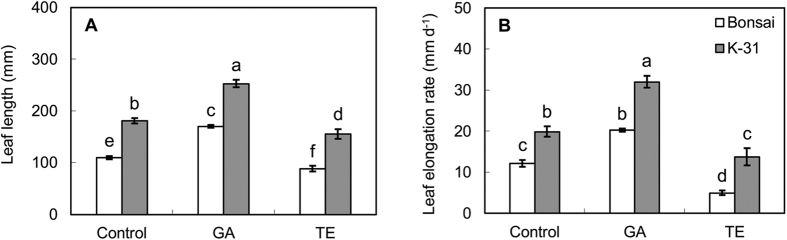
Leaf length (**A**) and leaf elongation rate (**B**) of the third leaf for ‘K-31’ and ‘Bonsai’ as affected by GA or TE treatment. The vertical bar is the standard error of mean leaf length (n = 4 replicates of GA or TE treatment, and each replicate contains at least 10 seedlings). Columns marked with different letters indicate significant differences between treatments and between cultivars based on LSD test (*P* = 0.05).

Exogenous application of GA significantly enhanced LER in both cultivars, with 61% and 66% greater leaf elongation rate in GA-treated leaves than untreated control leaves for ‘K-31’ and ‘Bonsai’, respectively (Fig. 6). In contrast, TE application inhibited leaf elongation in both cultivars, but to a greater extent for ‘Bonsai’ than ‘K-31’, with 31% reduction in leaf elongation rate of TE-treated ‘K-31’ and 60% reduction for ‘Bonsai’ (Fig. 6).

**Figure 7:**
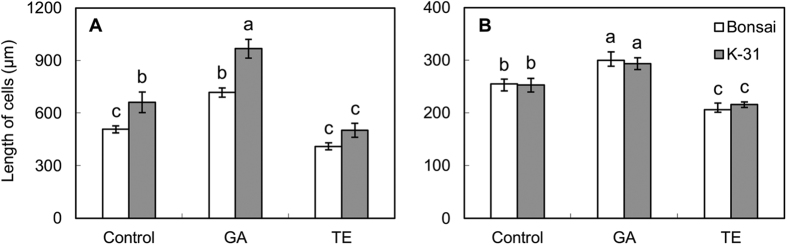
The length of cells on the abaxial epidermis of ‘K-31’ and ‘Bonsai’ as affected by GA or TE treatment. (**A**) Long cell; (**B**) Interstomatal cell. The vertical bar is the standard error of mean (n = 4 replicate of GA or TE treatment, and each replicate contains at least 10 seedlings). Columns marked with different letters indicate significant differences between treatments and between cultivars based on LSD test (*P* = 0.05).

**Figure 8:**
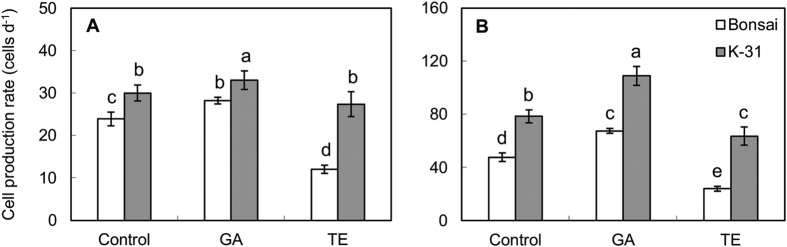
The production rates of cells on the abaxial epidermis of ‘K-31’ and ‘Bonsai’ as affected by GA or TE treatment. (**A**) Long cell; (**B**) Interstomatal cell. The vertical bar is the standard error of mean (n = 4 replicates of GA or TE treatment, and each replicate contains at least 10 seedlings). Columns marked with different letters indicate significant differences between treatments and between cultivars based on LSD test (*P* = 0.05).

In order to determine whether enhanced leaf elongation was due to increases in cell length and/or increases in cell production rate, the length of two types of epidermal cells and cell production rate of each cell type were compared between two cultivars with or without GA and TE treatments. The epidermal long cells in ‘K-31’ were longer than that in ‘Bonsai’ either with or without GA or TE application (Fig. 7A). However, the length of interstomatal cells did not differ between the two cultivars (Fig. 7B). The application of GA resulted in significant increases in epidermal long cells and interstomatal cells for both cultivars while TE inhibited cell length of both cell types in both cultivars. The cell production rate of both types of epidermal cells was significantly greater in ‘K-31’ than that in ‘Bonsai’ regardless of GA or TE treatments (Fig. 8A,B). Application of GA increased the production rate of both types of epidermal cells for both cultivars whereas TE treatment resulted in significant reduction in cell production rate for both epidermal types in “Bonsai’ leaves and only interstomatal cell production rate in ‘K-31’ leaves.

### Differential expression of expansin and XET between cultivars and responses to GA and TE treatment

**Figure 9:**
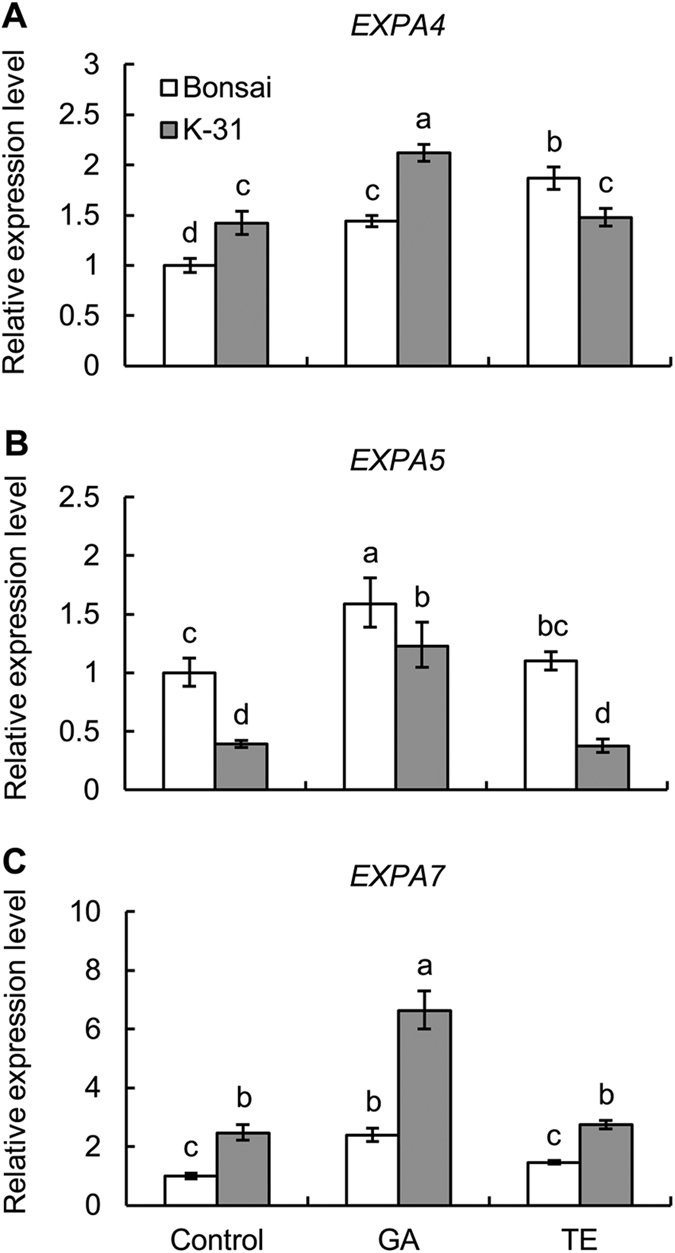
Relative gene expression level of α-expansin genes in leaves of ‘K-31’ and ‘Bonsai’ as affected by GA or TE treatment. (**A**) *EXPA4*; (**B**) *EXPA5*; (**C**) *EXPA7*. The vertical bar is the standard error of mean (n = 4 replicates of GA or TE treatment, and each replicate contains at least 10 seedlings). Columns marked with different letters indicate significant differences between treatments and between cultivars based on LSD test (*P* = 0.05).

**Figure 10:**
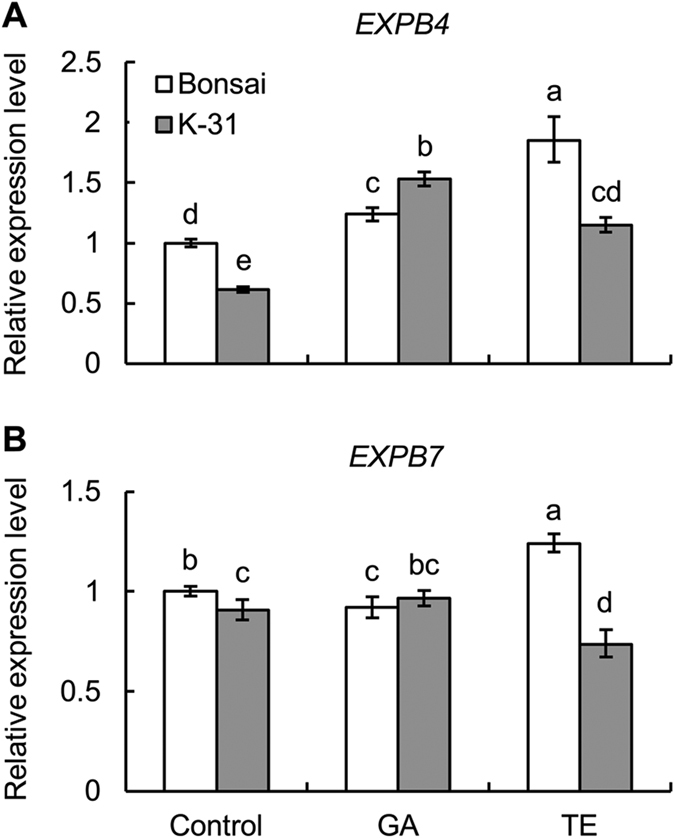
Relative gene expression level of β-expansin genes in leaves of ‘K-31’ and ‘Bonsai’ as affected by GA or TE treatment. (**A**) *EXPB4*; (**B**) *EXPB7*. The vertical bar is the standard error of mean (n = 4 replicates of GA or TE treatment, and each replicate contains at least 10 seedlings). Columns marked with different letters indicate significant differences between treatments and between cultivars based on LSD test (*P* = 0.05).

**Figure 11:**
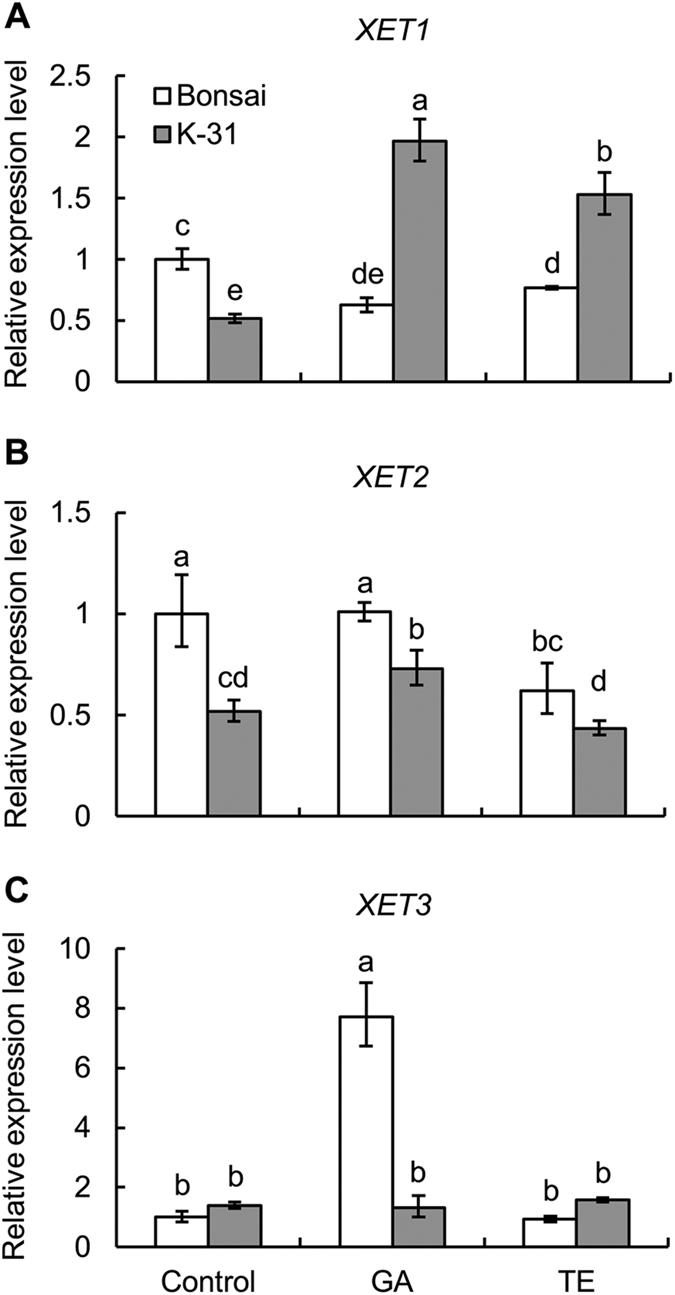
Relative gene expression level of XET genes in leaves of ‘K-31’ and ‘Bonsai’ as affected by GA or TE treatment. (**A**) *XET1*; (**B**) *XET2*; (**C**) *XET3*. The vertical bar is the standard error of mean (n = 4 replicates of GA or TE treatment, and each replicate contains at least 10 seedlings). Columns marked with different letters indicate significant differences between treatments and between cultivars based on LSD test (*P* = 0.05).

In order to determine whether the genetic variations and GA effects on leaf elongation are due to differences in the expression level of genes regulating cell elongation, transcript levels of several expansins and XET were analyzed. Five expansin ESTs (*EXPA4*, *EXPA5*, *EXPA7*, *EXPB4*, *EXPB7*) and three XET ESTs (*XET1*, *XET2*, *XET3*) were identified in tall fescue through EST search in NCBI database. Among the 5 expansin genes, 4 expansins including three α-expansins (*EXPA4*, *EXPA5* and *EXPA7*) and one β-expansin (*EXPB4*) were up-regulated by GA treatment in both cultivars, and only *EXPB7* was down-regulated by TE treatment in ‘K-31’ (Figs 9 and 10). Compared between the two cultivars, the expression level of *EXPA7* was significantly higher in ‘K-31’ than ‘Bonsai’ either with or without GA or TE treatment while *EXPA4* showed consistence differences between the two cultivars exposed to control and GA treatment. *EXPB7* and *XET1* neither show responses to GA treatment nor exhibit cultivar variations (Figs 10 and 11). The expression level of *XET2* and *XET3* genes did not show consistent differences between the two cultivars exposed to control, GA or TE treatment, but they exhibited differential responses to GA and TE treatment in both cultivars (Fig. 11). *XET1* and *XET2* expression increased with GA treatment for ‘K-31’ while it did not change in GA-treated ‘Bonsai’. *XET3* was not responsive to GA or TE treatment in ‘K-31’, but increased with GA treatment for ‘Bonsai’.

## Discussion

The leaf elongation rate and elongation duration time are two main factors contributing to the leaf growth. Here, the leaf elongation profiles of two tall fescue genotypes with contrasting elongation rates were examined. The leaf elongating duration times of ‘K-31’ and ‘Bonsai’ were similar (see Supplemental Fig. S2) and the leaf length differences between them were mostly due to the differences of leaf elongation rates. In a study of barley, *Rht3* dwarfing gene led the plants to a shorter leaf length compared with *rht3* wild type, along with reduced growth rate but same growth duration time^39^. *Rht3* mutant is generally supposed to loss the response to gibberellin, which is an important phytohormone greatly promoting the leaf elongation in many species, such as wheat^43^,^44^, barley^45^, and maize^46^.

The elongation zone is a sensitive factor influenced by genetic variations and GA regulation. Jovanovic *et al*.^47^ found in maize that the length of the growth zone in high-LER cultivar was 25% longer than that in low-LER cultivar^47^. Another study in wheat also reported that a GA-deficient dwarf mutant, M489, had reduced leaf length and maximum REGR and further experiments documented that exogenous gibberellic acid increased the leaf length and REGR of mutant plants up to the level of wild type. The results above inferred GA regulation might attribute to the genetic variation in leaf growth through controlling the length of leaf elongation zone and REGR. In this study, fast-growing ‘K-31’ leaves had longer (44%) leaf elongation zone than slow-growing ‘Bonsai’, and the maximum REGR of ‘K-31’ was significantly higher than ‘Bonsai’. Further, the endogenous GA content of two genotypes were examined and results indicated that the variations in LER between two tall fescue cultivars were associated with differential levels of endogenous GA_4_, with fast-growing ‘K-31’ leaves having greater GA_4_ content than slow-growing ‘Bonsai’ leaves.

Exogenous application of GA_3_ further stimulated LER in both cultivars. Similar results were found in leaves of soybeans (*Glycine max*) treated with different concentrations of GA_3_, which demonstrated that the increase of plant height with GA treatment was positively associated with the increased level of endogenous GA^48^. In our study, LER of ‘K-31’ was higher than ‘Bonsai’ after GA_3_ application, which was corresponded with the endogenous GA_1_ content. In addition, elongation of both interstomatal cells and long cells were stimulated by exogenous GA treatment and inhibited by TE treatment in our study, which suggested that GA could play regulatory roles in leaf elongation through controlling the cell elongation in tall fescue.

Variations in leaf elongation rate could also be due to differential elongation rates of different cell types. Tina *et al*. (2005) reported that the interstomatal cells in leaf blade had the same length between semi-dwarf and tall cultivars of wheat while the long cells in tall cultivars were much longer than the semi-dwarf cultivar^36^. Hu and Schmidhalter^49^ found that the reduction in leaf elongation in wheat by salinity stress was due to the inhibition of the long cell growth while the length of interstomatal cells remained unchanged under salinity stress^49^. In our study, interstomatal cell length did not differ between fast-growing ‘K-31’ and slow-growing ‘Bonsai’ whereas the long cells in ‘K-31’ were significantly longer than ‘Bonsai’, suggesting that the genetic variation in LER in tall fescue was largely associated with the differences in the growth rate of long cells, which could be manipulated through genetic modification generating fast- or slow-growing phenotypes.

Despite the knowledge of genetic variations and the well-known simulative effect of GA on leaf growth rate, few reports provide the information about the relationship between the GA response and expansin or XET genes expression in relation to the genetic variations in leaf growth. In our study, the expression levels of *EXPA7* were significantly higher in ‘K-31’ than ‘Bonsai’ with or without GA treatment, which inferred that the *EXPA7* was attributed to the higher leaf elongation rate of ‘K-31’ with their relaxation functions in the cell wall. Other expansin genes and XET genes tested in this study did not show consistent differences in their expression levels between the fast-growing and slow-growing cultivars, which might because the expansins and XETs are both families of genes, different members may play various roles in plant growth and development processes^50^,^51^. For instance, in a study with *Lycopersicon esculentum*, the expression of *LeEXP2* was positively associated with the elongation rate of hypocotyls, but the expression level of *LeEXP18* did not correlate with hypocotyl growth rate^52^.

GA effects on leaf elongation and genetic variations in leaf elongation could be due to the involvement of different cell-wall loosening genes. In our study, among five homologous expansin genes, three α-expansins (*EXPA4*, *EXPA5*, and *EXPA7*) and one β-expansin (*EXPB4*) were up-regulated by GA treatment in both cultivars, suggesting that those expansin genes could play roles in GA-enhanced cell elongation in tall fescue. The expression level of *EXPA5* was greatly up-regulated by GA treatment in both genotypes, suggesting that it could be induced by high GA level. In the high-LER genotype ‘K-31’, however, which had higher endogenous GA content than ‘Bonsai’, *EXPA5* expression level was lower than ‘Bonsai’ both with and without GA treatment. One explanation about this might be the gene *EXPA5* in the ‘K-31’ was not as sensitive to GA as it in ‘Bonsai’, due to less GA receptors or transcriptional factors.

Differential response patterns of XET genes were found between the two cultivars in response to GA, with *XET1* and *XET2* being up-regulated in ‘K-31’ and *XET3* up-regulated in ‘Bonsai’ with GA treatment. The results indicated that members of gene family are likely to play different roles in various plant genotypes within the same species. The results above together suggested that *EXPA4* and *EXPA7* could be more important accounting for the genetic variations in leaf cell elongation in tall fescue, while *EXPA4*, *EXPA5*, *EXPA7*, *EXPB4*, *XET1*, *XET2* and *XET3* could be regulated by GA, although multiple cell-wall loosening genes could coordinately regulate cell elongation controlled genetically or regulated by GA.

## Conclusions

Taken together, this study demonstrated that the genetic variation in leaf elongation of tall fescue cultivars with differential growth rate were associated with the differential leaf elongation zones differing in both cell elongation rate and production rate. Cultivar differences in endogenous GA content and exogenous treatment of plants with GA or TE suggested that GA could play roles in regulating leaf elongation in association with the up-regulation of several expansin and XET genes. Those cell-wall loosening genes, including *EXPA4* and *EXPA7*, related to genetic variations and responsive to GA (*EXPA4*, *EXPA5*, *EXPA7*, *EXPB4*, *XET1*, *XET2* and *XET3*), could be used as potential candidate genes to modify genetically grass leaves for rapid leaf elongation as needed in forage grass or slow leaf elongation as a desirable trait for turf grass through gene over-expression or knockout.

## Materials and Methods

### Plant materials and growth conditions

Seedlings of tall fescue (‘K-31’ and ‘Bonsai’) were established from seeds planted in plastic containers filled with fritted clay. Seedlings were watered daily and fertilized weekly with half-strength Hoagland’s nutrient solution^53^. Plants were maintained in a walk-in growth chamber (Environmental Growth Chambers, Chagrin Falls, OH) controlled at 22/18 °C (day/night) temperature, 60% relative humidity, and 12-h photoperiod with photosynthetically-active radiation of 750 μmol m^−2^s^−1^ at the canopy level. The experiments were conducted at Rutgers University between March and June in 2015. Grasses for the genetic variation analysis of LER and REGR were seeded on 15^th^, March, 2015 and the first leaf emerged mostly on 21^th^, March, which was recorded as the Day 1 of first leaf elongation. Most of second leaves appeared on 28^th^, March, which was recorded as the Day 1 of second leaf elongation, while the Day 1 of third leaf was 4^th^, April.

### Gibberellin (GA) and gibberellin inhibitor treatments

Grasses for GA regulation experiment were seeded on 1^st^, April, 2015 and the for the uniformity of GA treatments, a hydroponic system was used in a growth chamber as previous described^54^. Uniform size one-week old seedlings at 1.2 (Haun Index) leaf stage^55^ established under the above conditions were transferred (14^th^, April) to plastic containers (54 cm in length, 42 cm in width, and 14 cm in depth) containing modified Hoagland’s nutrient solution. The nutrient solution contained ammonium sulfate ((NH4)_2_SO_4_, 71.36 mg L^−1^), potassium nitrate (KNO_3_, 27.3 mg L^−1^), calcium nitrate tetrahydrate (Ca(NO_3_)_2_.4H_2_O, 120.8 mg L^−1^), potassium phosphate monobasic (KH_2_PO_4_, 81.65 mg L^−1^), potassium sulfate (K_2_SO_4_, 52.28 mg L^−1^), magnesium sulfate anhydrous (MgSO_4_, 60 mg L^−1^), EDTA, ferric sodium salt trihydrate (Fe(EDTA)Na, 16.84 mg L^−1^), boric acid (H_3_BO_3_, 1.43 mg L^−1^), manganese chloride (MnCl_2_.4H_2_O, 0.91 mg L^−1^), zinc sulfate (ZnSO_4_. H_2_O, 0.11 mg L^−1^), cupric sulfate (CuSO_4,_ 0.04 mg L^−1^), ammonium molybdate ((NH_4_)Mo_7_O_24_.4H_2_O, 0.01 mg L^−1^). The nutrient solution was aerated by air pumps (115 V, 60 Hz, Tetra Blacksburg, VA) and changed every 5 d. The pH of nutrient solution was adjusted every other day to 5.8 using KOH.

For the investigation of GA regulation of leaf elongation, seedlings were treated with GA_3_ (Sigma-Aldrich, St. Louis, MO) or a GA inhibitor, trinexapac-ethyl (TE) on 24^th^, April. For GA_3_ treatment, roots of plants was immersed in the nutrient solution with GA_3_ for 12 h, and leaf elongation rate was evaluated for 3 days after GA treatment. Different concentrations (0, 10, 25, 50, 100, and 200 μmol L^−1^) of GA_3_ were tested and the 50 μmol L^−1^ was found to be most effective in promoting leaf elongation and was selected to use in the subsequent experiment. For TE, the product Primo Maxx (Syngenta Professional Products, Greensboro, NC) was sprayed onto the leaves of grasses until dripping at the manufacture recommended concentration (2 mL L^−1^ [v/v]; a.i. TE = 11.3%) for tall fescue plants. The untreated control plants were sprayed with equal volume of water as used in TE treatment.

The experiment with GA or TE treatments for two cultivars were arranged in a split-plot design with GA or TE treatment as main plots (4 containers for each treatment) and cultivars as sub-plots (40 plants for each cultivar) which were randomly placed within each container with or without GA or TE treatment. The GA or TE treatment was repeated in four containers as four replicates. Each cultivar had 40 plants as replicates for each sampling of leaves for various measurements described below. Each plant was wrapped at the base of the plant with a sponge strip which was placed in a hole of a styrofoam plate, holding the plant in upright position in each container.

### Leaf elongation rate (LER) and relative elemental growth rate (REGR)

The length of the first three leaves of both plant cultivars (‘K-31’ and ‘Bonsai’) was monitored from the first day of the leaf emergence till the third leaves were fully expanded, and the leaf elongation rate (LER, mm d^−1^) for each leaf was calculated as the slope of the linear regression line through the data points within the phase of linear increase in leaf length. The linear growth phase of the leaves was determined as the interval between 20 and 80% of final leaf length^38^. If we described the function of leaf length (y) to days of leaf elongation (x) as equation (1) y = mx + b, the slope m was the LER of each leaf. The linear regression line was drawn by Excel with the data plots selected within the linear interval and the LER (m) was calculated by the equation (2) m = [n∑(xy) − ∑x∑y]/[n∑(x^2^) − (∑x)^2^], which x is the days of leaf elongation, y is the leaf length and n is the days of recorded. The R^2^ is the square of the correlation coefficient.

The length of the third leaf on each plant for each cultivar and GA_3_ or TE treatment was measured using a ruler every day, beginning when the leaf tip just emerged. The REGR was a series of segmental growth rates along elongating leaves and determined by the leaf length increment within 24 h^56^. The spatial distribution of leaf REGR was determined using a pinhole method^10^. As previous reports indicated, the spatial distribution of REGR was steady between the emergence of the leaf tip and the transition from blade to sheath growth^10^. Therefore, a set of 10 plants per replicate was selected when the third leaf shortly emerged. Holes were pinned with fine needles (0.25 mm diameter) through the sheaths of the outer leaves and the first hole is 2 mm from the leaf base. The elongation zone of tall fescue is restricted within the base region of the leaf, and 30 holes spaced 2 mm apart were pierced along the longitudinal axial of leaf. After 24 h, the third leaves were removed from the plant and observed under a stereomicroscope fitted with a camera (Nikon Instruments Inc., model SMZ1270, Melville, NY, USA). The final positions of the holes along the third leaves were recorded using the camera. The relative elemental growth rate (REGR, mm mm^−1^ h^−1^) was calculated as described by Arredondo^12^: REGR = (*d*_f_ − d_i_)/(*d*_i_ × Δ*t*) where *d*_f_ (mm) and *d*_i_ (mm) is the final and initial distances between two holes along the leaves and the Δ*t* (h) is the time period between pinning and observation.

### Epidermal cell length and cell production rate

The third leaves were harvested when they were fully expanded. Leaves were cut at the base of leaf blade and transferred into methanol immediately for chlorophyll removal. Then the leaves were transferred to 85% lactic acid for storage. The abaxial surface of leaf was brushed by nail polish and a fine transparent negative film of the epidermis was obtained. The picture of epidermis was observed under microscope and captured by a camera. The length of two kinds of epidermal cells (long cells and interstomatal cells) are measured by software Digimizer (MedCalc Software bvba, Ostend, Belgium) and compared among different treatments^36^. The characteristics of each type of epidermal cells were according to the previous report of Botwright^36^ in regard to the wheat leaf. The interstomatal cells are the subsidiary cells between two stomata and long cells are long, unspecialized cells between the two cell rows of interstomatal and guard cells (see Supplemental Fig. S1). Cell production rates of both kinds of epidermis cells were calculated based on the mature cell length (L_m_) and leaf elongation rate using the equation P = LER (mm d^−1^) ×L_m_ (mm)^−1^, assuming during the steady-state leaf elongation, the flux of cells through any point in the elongation-only zone is constant and represents the rate of cell production (P, cells d^−1^)^3^. Epidermal cell length and cell division rate were determined on 40 leaves from 40 plants for each cultivar subjected to GA or TE treatment. For each leaf, 50 cells were measured and the average length and cell production rate was taken to use for the further analysis.

### Quantification of GA content in leaves

Gibberellic acid extraction and quantification was based on the method used by^57^. Frozen tissue samples were lyophilized using VirTis Genesis freeze dryer (SP Scientific, model 12 EL, NY). Lyophilized samples was ground to a fine powder using Genogrinder 2000 (OPS Diagnostics, model SP2100-115, NJ) and approximately 50 mg was weighed and used for the hormone extraction and analysis. Extractions were handled in the same manner as described for kentucky bluegrass (Poa pratensis) in Krishnan and Merewitz (2014)^58^. 100 nmol of deuterium-labeled GA_4_ (d_2_-GA_4_) and GA_1_ (GA_1_) was added at the time of extraction as the internal standard for liquid chromatography (LC) analysis. GA content was analyzed using an Ultra High-performance Liquid Chromatography-tandem mass spectrometer (UPLC/MS/MS) (Waters Quattro Premier XE ACQUITY^®^ Tandem Quadrupole, Waters, Milford, MA).

### Gene expression analysis

The expression level of expansin and XET genes in leaves under different treatments were examined using qPCR (quantitative Polymerase Chain Reaction). The entire tall fescue EST (Expressed Sequence Tags) database in NCBI (National Center for Biotechnology Information, http://www.ncbi.nlm.nih.gov/) was searched using expansin and XET gene families of rice, wheat, meadow fescue and *Brachypodium distachyon* based on the similarity, and 5 expansin ESTs and 3 XET ESTs were found in the tall fescue EST database. Primers of 5 expansin ESTs and 3 XET genes for quantitative PCR were designed by Primer3 using those sequences which are listed Table 1. Because meadow fescue and tall fescue have very similar genetic background, the gene *PpXET3* of meadow fescue, which was not found to have a homologous sequence in tall fescue’s EST database, was used to design the qPCR primers along with the other EST sequences.

The third leaves of each plant were harvested and frozen in liquid nitrogen for the RNA extraction. Total RNA was extracted using Trizol reagent (Life Technologies, Grand Island, NY) and the contaminating DNA was removed by TURBO DNA-free kit (Life Technologies, Grand Island, NY). Reverse transcription of total RNA to single strand DNA was performed by the high capacity cDNA reverse transcription kit (Life Technologies, Grand Island, NY). The qPCR was performed in StepOne Real-time PCR System (Life Technologies, Grand Island, NY) and the cycling condition was 95 °C for 10 mins, 40 cycles of 95 °C denaturation for 30 seconds and 60 °C annealing/extension for 60 seconds, 95 °C for 30 seconds, followed by dissociation curve analysis. Power SYBR Green PCR Master Mix (Life Technologies, Grand Island, NY) was the intercalating dye used to detect gene expression level. Gene name, accession number, forward and reverse primer sequences are provided in Table 1. A tall fescue actin gene was used as the reference gene^59^ and a ΔΔCt method was used to calculate the relative expression level of interest and reference genes.

### Statistical analysis

All data were subjected to the analysis of variance test using the general linear model with a statistical program (SAS 9.0, Cary, NC). The differences between GA, TE and the untreated control treatments and cultivar variations in LER, cell elongation rate, cell production rate, and gene expression levels were also tested using the least significance test at probability level of 0.05.

## Additional Information

**How to cite this article**: Xu, Q. *et al*. Gibberellin-Regulation and Genetic Variations in Leaf Elongation for Tall Fescue in Association with Differential Gene Expression Controlling Cell Expansion. *Sci. Rep.* **6**, 30258; doi: 10.1038/srep30258 (2016).

## Supplementary Material

Supplementary Information

## Figures and Tables

**Table 1:** Gene name, accession number, forward and reverse primer sequences used in q-PCR analysis of gene expression in tall fescue.

Gene	GenBank	Primers (5′-3′)	
*EXPA4*	DT684026.1	forward	ATCGTGCCCGTCGCATAC
		reverse	TGACCAGCACCAGGTTGAAG
*EXPA5*	DT709329.1	forward	AGGGTGGCGTGCCAGAA
		reverse	TTGGTGACGAGGACGAGGTT
*EXPA7*	DT686661.1	forward	TGCCGTGCCGGAAGTC
		reverse	TGATCAGCACCAGGTTGAAGTAG
*EXPB4*	DT707038.1	forward	GGCAACCAGCCGCTGTT
		reverse	GAGCAAGCCTTGTGCTTCGT
*EXPB7*	DT710510.1	forward	CGGCATCATCGACATGCA
		reverse	ACCCGTGCTGTACGTGGAA
*XET1*	DT683504.1	forward	GCACCGTCACAGCCTACTACCT
		reverse	GGTCTCGTTGCCCAGGAA
*XET2*	DT707331.1	forward	GCCCTACGTGATGAACACCAA
		reverse	AGGGATCGAACCAGAGGTAGAAC
*XET3*	AJ295945.1	forward	CGTTGATTCCGGTGCTAGCT
		reverse	GTCGCAATCGTCGTTGAAGTT
*Actin*	AY194227.1	forward	TCTTACCGAGAGAGGTTACTCC
		reverse	CCAGCTCCTGTTCATAGTCAAG
