# Supplementary material for: Gibberellin-Regulation and Genetic Variations in Leaf Elongation for Tall Fescue in Association with Differential Gene Expression Controlling Cell Expansion
Source: Sci Rep. 2016 Jul 26;6:30258. doi: 10.1038/srep30258 (PMC4960529; doi:10.1038/srep30258)
Supplement: Supplementary Information [file srep30258-s1.pdf]

Gibberellin-Regulation and Genetic Variations in Leaf Elongation for Tall Fescue in Association with Differential Gene Expression Controlling Cell Expansion

Qian Xu[1,2], Sanalkumar Krishnan[3], Emily Merewitz[3], Jichen Xu[1*], Bingru Huang[2*]

[1]National Engineering Laboratory for Tree Breeding, College of Biological Sciences and Technology, Beijing Forestry University, Beijing, China,100083

[2]Department of Plant Biology and Pathology, Rutgers University, New Brunswick, NJ, United States of America, 08901

[3]Department of Crop Science, Michigan State University, East Lansing, MI, United States of America, 48824

* E-mails of the corresponding authors:

Bingru Huang: huang@aesop.rutgers.edu

Jichen Xu: jcxu282@sina.com

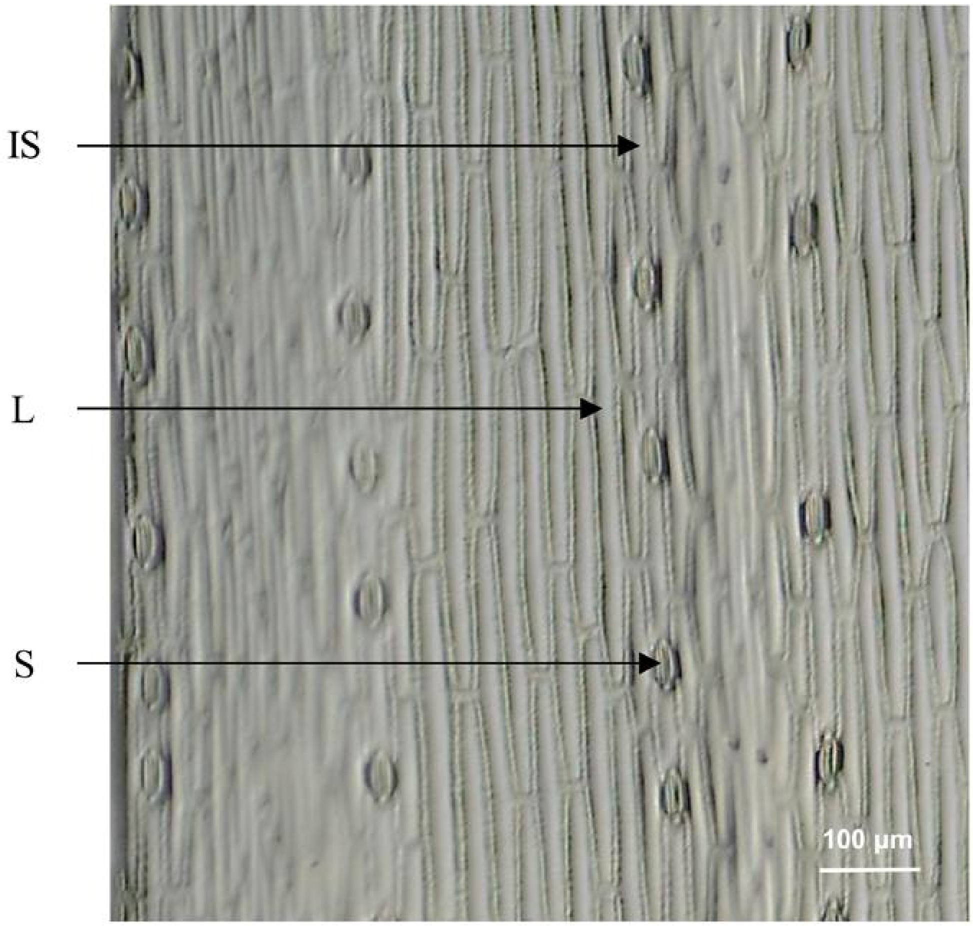

Supplemental Figure S1. The abaxial leaf surface of tall fescue, showing the two cell types measured in this study. L, long cells; IS, interstomatal cells; S, stoma.

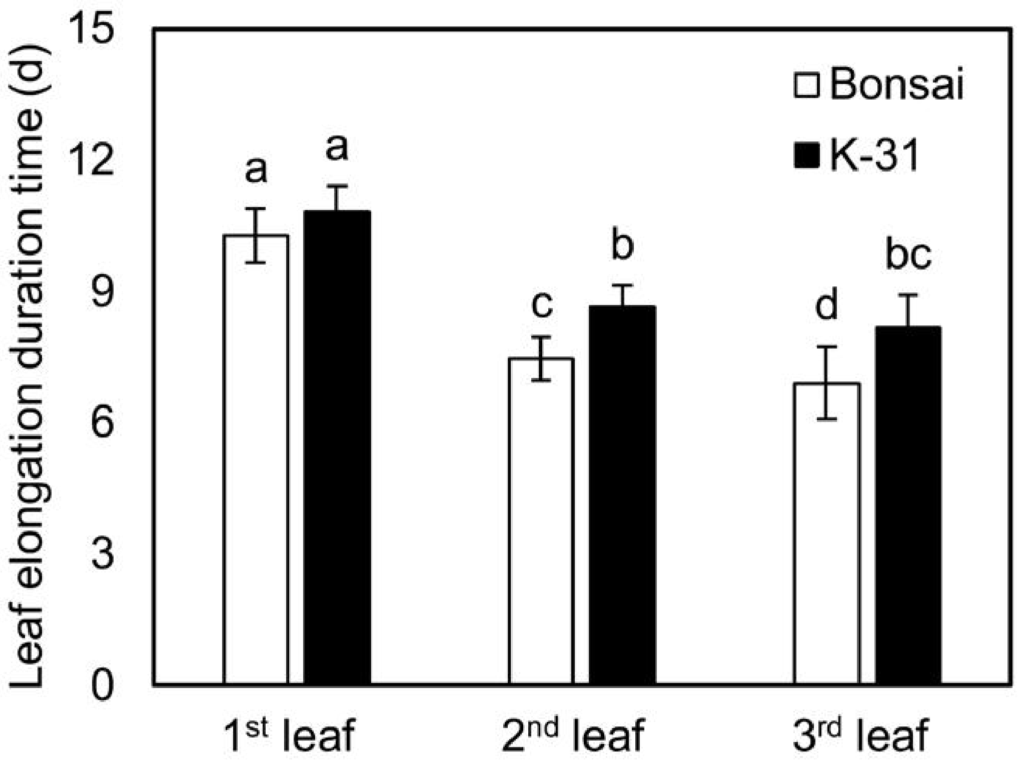

Supplemental Figure S2. The leaf elongating duration times of both tall fescue genotypes, ‘K-31’ and ‘Bonsai’. The first three leaves were included. The vertical bar is the standard error of mean (n = 10 seedlings for each genotype). Columns marked with different letters indicate significant differences between cultivars based on LSD test (P = 0.05).
